# Celastrol protects mouse retinas from bright light-induced degeneration through inhibition of oxidative stress and inflammation

**DOI:** 10.1186/s12974-016-0516-8

**Published:** 2016-02-27

**Authors:** Minjuan Bian, Xiaoye Du, Jingang Cui, Peiwei Wang, Wenjian Wang, Weiliang Zhu, Teng Zhang, Yu Chen

**Affiliations:** Clinical Research Institute of Integrative Medicine, Shanghai University of Traditional Chinese Medicine, Shanghai, 200437 China; Yueyang Hospital, Shanghai University of Traditional Chinese Medicine, 110 Ganhe Rd, Shanghai, 200437 China; Shanghai Institute of Materia Medica, Shanghai, 201203 China

## Abstract

**Background:**

Photoreceptor death leads to vision impairment in several retinal degenerative disorders. Therapies protecting photoreceptor from degeneration remain to be developed. Anti-inflammation, anti-oxidative stress, and neuroprotective effects of celastrol have been demonstrated in a variety of disease models. The current study aimed to investigate the photoreceptor protective effect of celastrol.

**Methods:**

Bright light-induced retinal degeneration in BALB/c mice was used, and morphological, functional, and molecular changes of retina were evaluated in the absence and presence of celastrol treatment.

**Results:**

Significant morphological and functional protection was observed as a result of celastrol treatment in bright light-exposed BALB/c mice. Celastrol treatment resulted in suppression of cell death in photoreceptor cells, alleviation of oxidative stress in the retinal pigment epithelium and photoreceptors, downregulation of retinal expression of proinflammatory genes, and suppression of microglia activation and gliosis in the retina. Additionally, leukostasis was found to be induced in the retinal vasculature in light-exposed BALB/c mice, which was significantly attenuated by celastrol treatment. In vitro, celastrol attenuated all-*trans*-retinal-induced oxidative stress in cultured APRE19 cells. Moreover, celastrol treatment significantly suppressed lipopolysaccharides-stimulated expression of proinflammatory genes in both APRE19 and RAW264.7 cells.

**Conclusions:**

The results demonstrated for the first time that celastrol prevents against light-induced retinal degeneration through inhibition of retinal oxidative stress and inflammation.

**Electronic supplementary material:**

The online version of this article (doi:10.1186/s12974-016-0516-8) contains supplementary material, which is available to authorized users.

## Background

Photoreceptor cell death plays important role in the pathogenesis of vision impairment in several retinal degenerative disorders, for instance, retinitis pigmentosa (RP), Stargardt disease, and age-related macular degeneration (AMD) [1, 2]. Light damage to the retina is causally associated with human retinal degeneration [3]. Light-induced retinal degeneration in rodents is primarily characterized by apoptotic photoreceptor cell death, mimicking clinical pathologies of human retinal disorders [4]. Thus, animal model of light-induced retinal degeneration is widely adopted to investigate retinal protective therapies against the loss of photoreceptors. To date, no effective photoreceptor protective therapies are clinically available yet, and therapeutic development targeting photoreceptor cell death is required for optimal vision preservation.

Oxidative stress is causally associated with cell death through multiple mechanisms and is regarded as one of the central players in the pathogenesis of various retinal degenerative disorders [5–8]. Accumulated evidence has also supported an important role of inflammation in the pathogenesis of retinal degenerative disorders [9–11]. Celastrol is a naturally occurring quinone methide triterpene present in Celastraceae family herbs that have a long history of usage in traditional Chinese medicine to treat chronic inflammation and autoimmune diseases in patients [12]. In addition to the effects on inflammation, autoimmune disorders, cancer, and obesity [13–16], the neuroprotective effect of celastrol in part implicating the mechanism of anti-oxidative stress has also been reported in a number of models [17–20]. A recent study has also reported that celastrol protects ganglion cells (GC) from optic nerve crush-induced damage through inhibiting retinal expression of TNF-α [21]. However, whether celastrol could protect photoreceptors from degeneration remains to be addressed.

In the current study, the effect of celastrol on light-induced retinal degeneration was evaluated in BALB/c mice. The results revealed significant morphological and functional protection of celastrol against bright light-induced retinal damage. The results also demonstrated that bright light caused prominent oxidative stress in retinal pigment epithelium (RPE), enhanced retinal expression of proinflammatory genes, microglial activation, and retinal gliosis and leukostasis in retinal vasculature, which were significantly inhibited by celastrol treatment.

## Methods

### Animals

Four- to 5-week-old female BALB/c mice were purchased from Shanghai Laboratory Animal Research Center. Mice were housed in a 12/12-h light-dark cycle room with temperature of 25 ± 2 °C. For bright light exposure experiments, mice were dark-adapted for 24 h prior to white light exposure (compact fluorescence lamp, 45 W, Chaoya Lighting, Shanghai, China) at 5000 lx for 2 h and 10,000 lx for 2 h or 30 min. All the animal handling procedures were reviewed and approved by the Institutional Animal Care and Use Committee of Shanghai University of TCM and carried out in adherence to the ARVO Statement for the Use of Animals in Ophthalmic and Vision Research.

### Chemicals

Celastrol was purchased from Sigma-Aldrich (USA), dissolved in DMSO, and administered to mice 30 min prior to light exposure via intraperitoneal injection (i.p). Mice unexposed to bright light and light-exposed mice without celastrol treatment received DMSO injection only. All-*trans*-retinal (atRAL) and lipopolysaccharides (LPS) were obtained from Sigma-Aldrich (USA).

### Cell culture

ARPE19 cells were purchased from the American Type Culture Collection (ATCC) and cultured in Dulbecco’s modified Eagle medium: Nutrient Mixture F-12 (DMEM/F-12) (Gibco, Thermo Fisher Scientific, USA) supplemented with 10 % fetal bovine serum, 50 μg/ml streptomycin, and 50 U/ml penicillin (Gibco, Thermo Fisher Scientific, USA). RAW 264.7 cells were obtained from Shanghai Institute of Biological Science (SIBS) and grown in Dulbecco’s modified Eagle medium (DMEM) (Gibco, Thermo Fisher Scientific, USA) with 10 % fetal bovine serum, 50 μg/ml streptomycin, and 50 U/ml penicillin (Gibco, Thermo Fisher Scientific, USA).

### LPS stimulation

After pretreatment with celastrol at indicated concentrations for 30 min, ARPE19 or RAW 264.7 cells were stimulated with LPS at concentrations of 1 μg/ml and 5 ng/ml, respectively. Cells were then harvested for RNA extraction 6 h after LPS treatment.

### In vitro detection of reactive oxygen species (ROS)

For in vitro ROS detection, ARPE19 cells were pretreated with celastrol at indicated concentrations for 30 min prior to incubation of atRAL at 20 μM. ROS probe 2′,7′-dichlorofluorescein diacetate (DCF-DA) (Sigma-Aldrich, USA) was then added to cells at 400 nM and incubated at 37 °C for 10 min. ROS signal from various treatments was obtained at the same setting using IncuCyte ZOOM (ESSEN Bioscience, USA). Fluorescence quantification was performed with IncuCyte ZOOM software using of the setting of green fluorescence integrated intensity. Relative fluorescence intensities were calculated for statistical analyses.

### Optical coherence tomography (OCT)

OCT (Optoprobe, Canada) was performed after anesthesia of mice by pelltobarbitalum natricum, i.p, at the dose of 65 mg/kg bw. Pupils were dilated by 1 % tropicamide prior to OCT imaging.

### Histology and immunohistochemistry (IHC)

Eyes were enucleated, fixed in 4 % paraformaldehyde, and processed for paraffin embedding. Paraffin sections 4 μm thick were subjected to hematoxylin and eosin (H&E) staining and measurement of the thickness of outer nuclear layer (ONL). For IHC, cryosections 12 um thick were incubated with primary antibodies including mouse anti-Rhodopsin (1: 2000, Novusbio, USA), rabbit anti-opsin M (1: 100, Millipore, USA), goat anti-GFAP (1:200, Abcam, USA), rabbit anti-vimentin (1:50, Cell Signaling Technology, USA), rabbit anti-Iba1 (1:500, Wako, Japan), or rabbit anti-COX2 (1:100, Abcam, USA), which was followed by incubation of secondary antibodies including Cy3-conjugated sheep anti-mouse, sheep anti-rabbit, or rabbit anti-goat secondary antibodies (1: 1000, Sigma-Aldrich, USA). 4-6-Diamidino-2-phenylindole (DAPI) staining was performed for nuclei visualization and measurement of the thickness of outer nuclear layer (ONL). Images were observed under fluorescent microscope (DM6000B, Leica, Germany).

### Electroretinogram (ERG)

Under dim red light, the dark-adapted mice were anesthetized with a mixture of ketamine hydrochloride (82.5 mg/kg bw) and xylazine (8.25 mg/kg bw). Scotopic ERGs were generated with flashes of green light at intensities ranging from −2 log cd s m^−2^ to 3.1 log cd s m^−2^. Five recordings were made at sufficient intervals between flash stimuli (from 5 s to 1 min) to allow recovery from any photobleaching effects. ERG was recorded and analyzed with the universal testing and electrophysiological system, Ganzfeld (Phoenix Research labs, USA).

### TdT-mediated dUTP nick-end labeling (TUNEL) assay

Cryosections 8 μm thick were used for TUNEL staining following the manufacturer’s instructions (DeadEnd™ Fluorometric TUNEL system, Promega, USA). Images were observed under fluorescent microscope (DM6000B, Leica, Germany).

### Real-time PCR analysis

Total RNA extraction of mouse retinas, ARPE 19, and RAW 264.7 cells was performed using TRIzol reagent (Invitrogen, USA). Reverse transcription was performed using PrimeScript RT Master Mix (TaKaRa, Japan) and real-time PCR was performed using QuantiTect SYBR Green PCR Master Mix (Qiagen, USA) on Roche LightCycler 480 II. All samples were run in triplicates; fold changes of the expression of genes were calculated according to 2^−[Ct(target gene)−Ct(Gapdh)]^. The primer sequences were listed in the Table 1.

**Table 1:** Primer sequences

Gene	Forward primer	Reverse primer
Mouse IL1β	TGCCACCTTTTGACAGTGATG	AAGGTCCACGGGAAAGACAC
Mouse Ccl2	AGCTGTAGTTTTTGTCACCAAGC	GTGCTGAAGACCTTAGGGCA
Mouse COX2	CCGTACACATCATTTGAAGAACTTA	CTACCATGGTCTCCCCAAAGAT
Mouse TNFα	ACGTCGTAGCAAACCACCAA	GCAGCCTTGTCCCTTGAAGA
Mouse ICAM-1	TCCGGACTTTCGATCTTCCAGCTAC	CCAGGTATATCCGAGCTTCAGAGGC
Mouse VCAM-1	AAGAAAGGGAGACTGTCAAAGAACT	AACTTCATTATCTAACTTCCTGCCC
Mouse VEGF	GTACTTGCAGATGTGACAAGCCA	GGTGACATGGTTAATCGGTCTTT
Mouse GAPDH	CCGGTGCTGAGTATGTCGT	CCTTTTGGCTCCACCCTTC
Human IL1β	TTATTACAGTGGCAATGAGGATGAC	GGAAGGAGCACTTCATCTGTTTAG
Human Ccl2	CTCATAGCAGCCACCTTCATTC	CTCTGCACTGAGATCTTCCTATTG
Human COX2	GATTTGACCAGTATAAGTGCGATTG	GTTTGGAGTGGGTTTCAGAAATAAT
Human TNFα	CCTCTCTCTAATCAGCCCTCTG	CTACAACATGGGCTACAGGCTT
Human ICAM-1	AAGATAGCCAACCAATGTGCTAT	AAGATAGCCAACCAATGTGCTAT
Human GAPDH	ACTCTGGTAAAGTGGATATTGTTGC	GGAATCATATTGGAACATGTAAACC

### In vivo detection of ROS

Dihydroethidium (DHE) (Sigma-Aldrich, USA) was administered to the mice, i.p, at a dose of 20 mg/kg bw 2 h prior to euthanization. Cryosections 12 μm thick were subject to assessment of ROS signal under fluorescent microscope (DM6000B, Leica, Germany).

### Leukostasis assay

Intracardial perfusion was performed using 50 ml of 0.9 % saline solution, followed by perfusion of fluorescein-conjugated concanavalin A (ConA) (Vector Laboratories, USA) at the dose of 6.25 mg/kg bw. After ConA perfusion, unbound ConA was flushed out by perfusion with 50 ml of 0.9 % saline solution. Retinal flatmounts were examined under fluorescent microscope (DM6000B, Leica, Germany).

### Statistical analysis

All results were expressed as mean ± standard error of mean (S.E.M.). Statistical analyses were performed using independent-samples *T* test (SPSS 18, USA). Differences were considered statistically significant if *p* values <0.05.

## Results

### Celastrol protected retinas against bright light-induced photoreceptor degeneration

BALB/c mice were first exposed to bright light at the intensity of 5000 lx for 2 h, and celastrol was administered 30 min prior to light exposure at the dose of 1, 2.5, and 5 mg/kg bw, respectively. OCT imaging was performed 7 days after light exposure to evaluate retinal structures. As shown in Additional file 1: Figure S1a, compared to that from the mice unexposed to bright light, severely disrupted photoreceptor structure was observed in light-exposed DMSO-treated mice, which was primarily characterized by diminished ONL in the retina. In contrast, dose-dependent preservation of ONL was observed in celastrol-treated mice. Partial retinal protection could be observed when celastrol was administered at the dose of 2.5 mg/kg bw and improved protection was observed when celastrol was given at 5 mg/kg bw. Similar protection of celastrol treatment was observed when BALB/c mice were exposed to light at 10,000 lx for 30 min (Additional file 1: Figure S1b). To further evaluate the retinal protection of celastrol, light exposure was delivered at 10,000 lx for 2 h. As shown in Additional file 1: Figure S1c, no protection was observed when celastrol was administered at the dose of 1 and 2.5 mg/kg bw, respectively; however, remarkable protection was observed in the mice that received celastrol at the dose of 5 mg/kg bw.

**Fig. 1:**
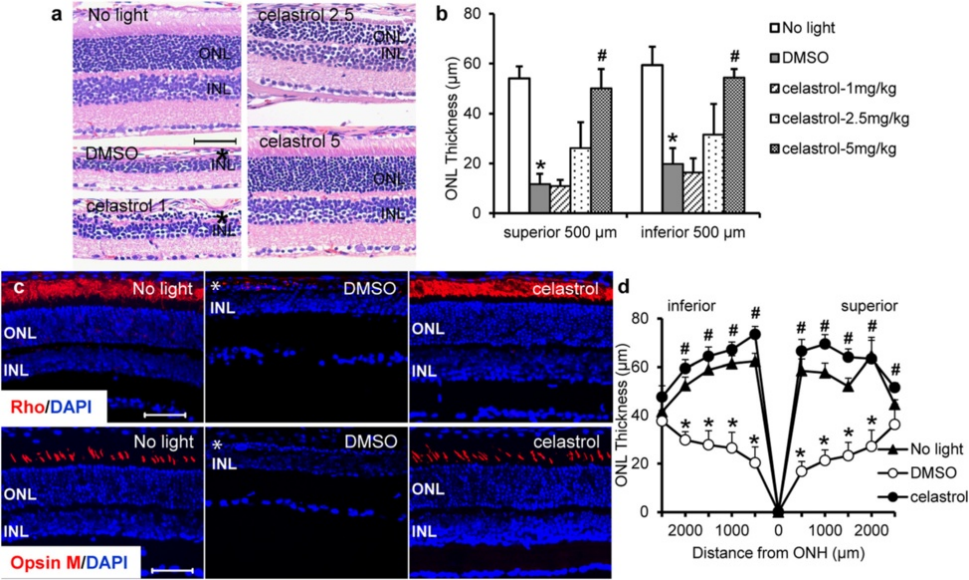
Celastrol preserved photoreceptor morphology in light-exposed BALB/c mice. Dark-adapted BALB/c mice were exposed to light at the intensity of 10,000 lx for 2 h after pretreatment with either vehicle control (*DMSO*) or celastrol at 1 mg/kg bw (*celastrol 1*), 2.5 mg/kg bw (*celastrol 2.5*), and 5 mg/kg bw (*celastrol 5*). **a** Eyes were enucleated 7 days after light exposure, and paraffin sections were made and stained with H&E for retinal histological examination using light microscopy. **b** ONL thickness was quantified at 500 μm off optical nerve head in both superior and inferior retina. **c** Dark-adapted BALB/c mice were exposed to light at the intensity of 10,000 lx for 2 h after pretreatment with either DMSO or celastrol at 5 mg/kg bw. IHC examination of rhodopsin (*Rho*) and opsin M (in *red*) expression in the central retinas (1000 μm off ONH) were performed 7 days after light exposure along with DAPI counterstaining (in *blue*) in the cryosections. **d** The thickness of ONL was measured in DAPI-stained cryosections. *Asterisk* indicates disorganized and reduced length of outer/inner segments and diminished ONL. *ONH* optic nerve head, *ONL* outer nuclear layer, *INL* inner nuclear layer. *Scale bar*: 50 μm. Data were expressed as mean ± S.E.M. (*n* = 4–6 per group). *Compared to that from no light, *p* < 0.05; ^#^compared to that from DMSO, *p* < 0.05

Histological examination of retinal gross histology and quantification of photoreceptor changes were further performed. Compared to that from the mice unexposed to bright light, prominent retinal morphological disruption was observed in DMSO-treated mice exposed to light at 5000 lx for 2 h (Additional file 1: Figure S2a and c), 10,000 lx for 30 min (Additional file 1: Figure S2b and d), or 10,000 lx for 2 h (Fig. 1a, b), which was characterized by disrupted and diminished photoreceptor outer segment (OS) and inner segments (IS) and reduced thickness of the ONL. In distinct contrast, when light was applied at 5000 lx for 2 h (Additional file 1: Figure S2a and c) or 10,000 lx for 30 min (Additional file 1: Figure S2b and d), partial protection from the loss of ONL was observed in the mice that were treated by celastrol at the dose of 2.5 mg/kg bw and an improved protection was observed in the mice treated by celastrol at the dose of 5 mg/kg bw. When light were applied at 10,000 lx for 2 h, significant retinal protection was observed in mice that were treated by celastrol at 5 mg/kg bw (Fig. 1a, b).

Retinal expression of rhodopsin (Rho) and mid-wavelength sensitive cone opsin (opsin M) was also examined. As shown in Fig. 1c and Additional file 1: Figure S3, compared to the abundant expression pattern of Rho and opsin M throughout the retinas from the mice unexposed to bright light, residual expression of these photoreceptor markers was observed in central retinas but not peripheral retinas of DMSO-treated mice exposed to light at 10,000 lx for 2 h. In contrast, well-organized and abundant expression of Rho and opsin M was readily observed in central retinas of light-exposed mice treated by celastrol at 5 mg/kg bw. Quantification of ONL thickness after DAPI staining showed that significant reduction in the thickness of ONL from light-exposed DMSO-treated mice compared to that from the mice unexposed to bright light, whereas the ONL thickness was significantly preserved in the retinas from light-exposed celastrol-treated mice (Fig. 1d). These results collectively indicated significant morphological protection of celastrol against light-induced photoreceptor degeneration in BALB/c mice.

### Celastrol protected retinas against bright light-induced functional impairment in BALB/c mice

**Fig. 2:**
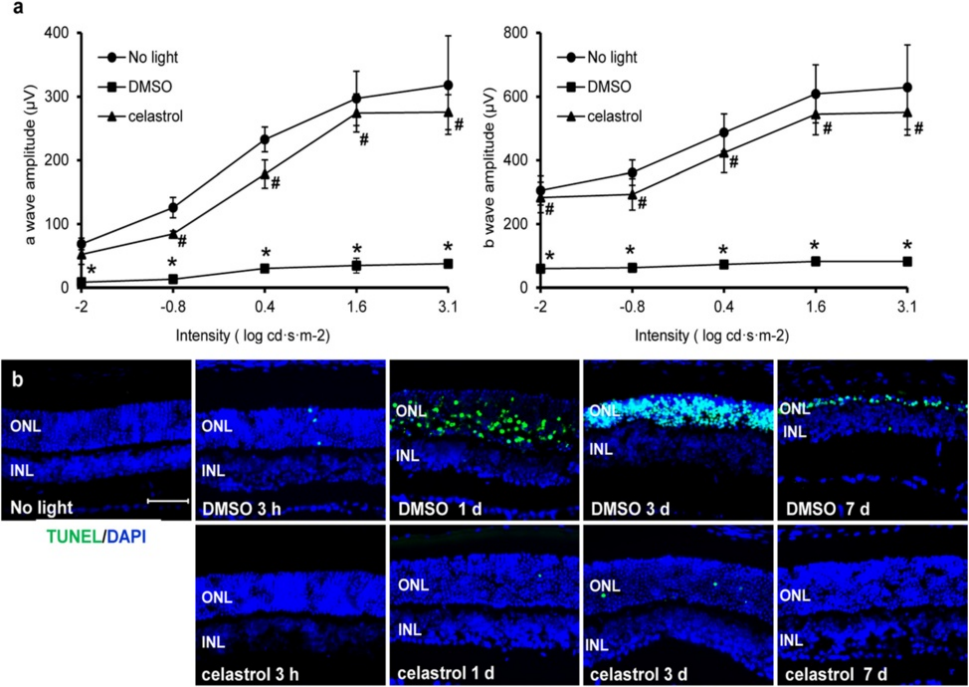
Celastrol preserved retinal function and protected against photoreceptor apoptosis in light-exposed BALB/c mice. **a** Dark-adapted BALB/c mice were exposed to light at the intensity of 10,000 lx for 30 min after pretreatment with DMSO and celastrol at 5 mg/kg bw. Retinal function was examined by ERG 4 days after light exposure. Scotopic ERG was recorded and both a-waves and b-waves were plotted to evaluate retinal function. Data were expressed as mean ± S.E.M. (*n* = 4 per group). *Compared to that from no light, *p <* 0.05; ^#^compared to DMSO, *p <* 0.05. **b** Dark-adapted BALB/c mice were exposed to light at 10,000 lx for 2 h after pretreatment with either DMSO or celastrol. Cryosections were made from eye cups collected from mice 3 h, 1 day, 3 days, and 7 days after light exposure along with those from the mice without light exposure (*no light*). Apoptosis was then examined by TUNEL staining (in *green*) and DAPI counterstaining (in *blue*) (*n* = 3–5 per group). *ONL* outer nuclear layer, *INL* inner nuclear layer. *Scale bar*: 50 μm

ERG was further performed to evaluate the retinal function. As shown in Fig. 2a, compared to that from the mice unexposed to bright light, significant reduction in scotopic a-wave and b-wave amplitudes was observed in DMSO-treated mice exposed to bright light at 10,000 lx for 30 min. However, significantly increased scotopic a-wave and b-wave amplitudes were observed in light-exposed mice treated by celastrol at 5 mg/kg bw compared to that from DMSO-treated mice. These results supported functional protection of retinas against bright light-induced degeneration as a result of celastrol treatment.

### Celastrol attenuated light-induced photoreceptor apoptosis

To further characterize the retinal protective effect of celastrol, apoptosis was examined by TUNEL assay at different time points after bright light exposure. As shown in Fig. 2b, no TUNEL-positive cells were found in the retinas from the mice unexposed to bright light. A few TUNEL-positive cells were detected in the ONL of retinas from DMSO-treated mice 3 h after bright light exposure at 10,000 lx for 2 h and TUNEL positivity peaked at 3 days after light exposure prior to massive clearance of photoreceptor cells by 7 days. At 7 days post light exposure, the remaining photoreceptor cells in the retinas were predominantly TUNEL positive. In distinct contrast, retinas from mice treated by celastrol at 5 mg/kg bw were characterized by remarkably decreased number of TUNEL-positive cells in the ONL. These results indicated that celastrol treatment protected photoreceptor cells from light-induced apoptosis.

### Celastrol alleviated light-induced oxidative stress in the retinas

**Fig. 3:**
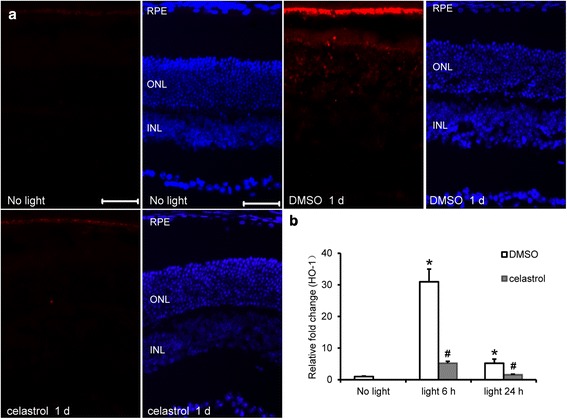
Celastrol suppressed retinal oxidative stress in light-exposed BALB/c mice. **a** Dark-adapted BALB/c mice were exposed to light at 10,000 lx for 2 h after pretreatment with either DMSO or celastrol at 5 mg/kg bw. Retinal ROS production was examined by DHE probe 1 day after light exposure in light-exposed mice and the mice without light exposure (*no light*). DAPI staining (in *blue*) and ROS signals (in *red*) were examined and recorded by fluorescence microscope (*n* = 4 per group). *RPE* retinal pigment epithelium, *ONL* outer nuclear layer, *INL* inner nuclear layer. *Scale bar*: 50 μm. **b** Retinas were collected 6 h and 1 day after light exposure and real-time PCR analyses of the expression of HO-1 was performed. Relative fold change against that from the mice without light exposure (*no light*) was presented. Data were expressed as mean ± S.E.M. (*n* = 4–6 per group). *Compared to that from no light, *p <* 0.05; ^#^compared to that from DMSO, *p <* 0.05

Retinal oxidative stress was further evaluated by examining in situ ROS production. As shown in Additional file 1: Figure S4, no visible ROS signal was detected in the retinas from the mice unexposed to bright light. In contrast, ROS production was remarkably increased in the retinal pigment epithelium (RPE) from DMSO-treated mice exposed to light at 10,000 lx for 2 h. The increase in ROS production in RPE was observed at 3 and 6 h after light exposure and persistent until 7 days after light exposure. It was also noted that the ROS signal was most prominent in the RPE 1 day after light exposure and in the meantime, ROS signals was detectable in the ONL in light-exposed DMSO-treated mice. Therefore, retinal ROS production was examined in light-exposed celastrol-treated mice 1 day after bright light exposure. As shown in Fig. 3a, remarkably decreased ROS signal in the RPE was observed in light-exposed celastrol-treated mice 1 day after light exposure. Moreover, ROS signal was not detected in the ONL from light-exposed celastrol-treated mice (Fig. 3a). The retinal expression of heme oxygenase 1 (HO-1) whose induced expression is indicative of light-induced oxidative stress in the retina [22] was also analyzed. As shown in Fig. 3b, compared to that from the mice unexposed to bright light, significantly increased expression of HO-1 was observed in the retinas of light-exposed DMSO-treated mice both 6 h and 1 day after bright light exposure. Celastrol treatment resulted in significantly reduced expression of HO-1 both at 6 h and 1 day after light exposure. These results demonstrated that celastrol protected retinas against light-induced oxidative stress.

### Celastrol attenuated light-induced expression of proinflammatory genes in the retina

**Fig. 4:**
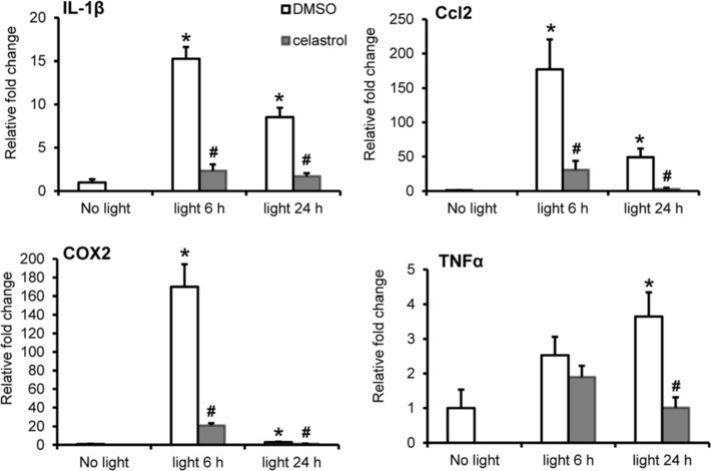
Celastrol attenuated the expression of proinflammatory genes in light-exposed retinas. Dark-adapted BALB/c mice were exposed to bright light at 10,000 lx for 2 h after pretreatment with either DMSO or celastrol at 5 mg/kg bw. Retinas were collected 6 h and 1 day after light exposure and further processed for total RNA isolation, reverse transcription, and real-time PCR analyses for expression of IL1β, Ccl2, COX2, and TNFα, respectively. After normalizing the expression to GAPDH, relative fold change of expression was calculated against that from the retinas collected from the mice unexposed to bright light (*no light*). Data were expressed as mean ± S.E.M. (*n* = 4–6 per group). *Compared to that from no light, *p <* 0.05; ^#^compared to that from DMSO, *p <* 0.05

**Fig. 5:**
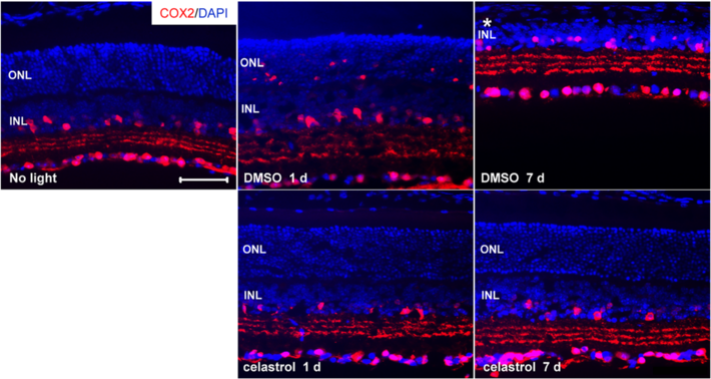
Celastrol suppressed light-induced expression of COX2 in photoreceptors. Dark-adapted BALB/c mice were exposed to light at 10,000 lx for 2 h after pretreatment with either DMSO or celastrol at 5 mg/kg bw. Eyes cups were made from enucleated eyes collected 1 and 7 days after light exposure. Cryosections were subject to IHC examination of COX2 expression (in *red*) and counterstained by DAPI (in *blue*) (*n* = 3–5 per group). *ONL* outer nuclear layer, *INL* inner nuclear layer. *Scale bar*: 50 μm

The retinal expression of proinflammatory and chemotactic cytokines was further examined, which included interleukin 1β (IL1β) [23], chemokine (C-C motif) ligand 2 (Ccl2) [24], cyclooxygenase-2 (COX2) [25], and TNFα [26]. As shown in Fig. 4, compared to that from the mice unexposed to bright light, bright light exposure at 10,000 lx for 2 h resulted in significantly increased expression of IL1β, Ccl2, and COX2 at 6 h and 1 day after light exposure in DMSO-treated mice. Increased expression of TNFα was not observed at 6 h but seen 1 day after light exposure. In contrast, compared to that from light-exposed DMSO-treated mice, significantly decreased expression of IL1β, Ccl2, COX2, and TNF-α was observed in the retinas from light-exposed celastrol-treated mice. IHC examination of retinal expression of COX2 was then performed. As shown in Fig. 5, expression of COX2 was observed in the GC, inner plexiform layer (IPL), and inner nuclear layer (INL) in the mice unexposed to bright light. In the retinas from light-exposed DMSO-treated mice, the immunoreactivity of COX2 was also detected in the ONL 1 day after light exposure at 10,000 lx for 2 h, which was not observed in the retinas from light-exposed celastrol-treated mice. These results provided evidence supporting that celastrol alleviated light-induced retinal inflammation.

### Celastrol inhibited leukostasis in the retinal vasculatures in bright light-exposed mice

**Fig. 6:**
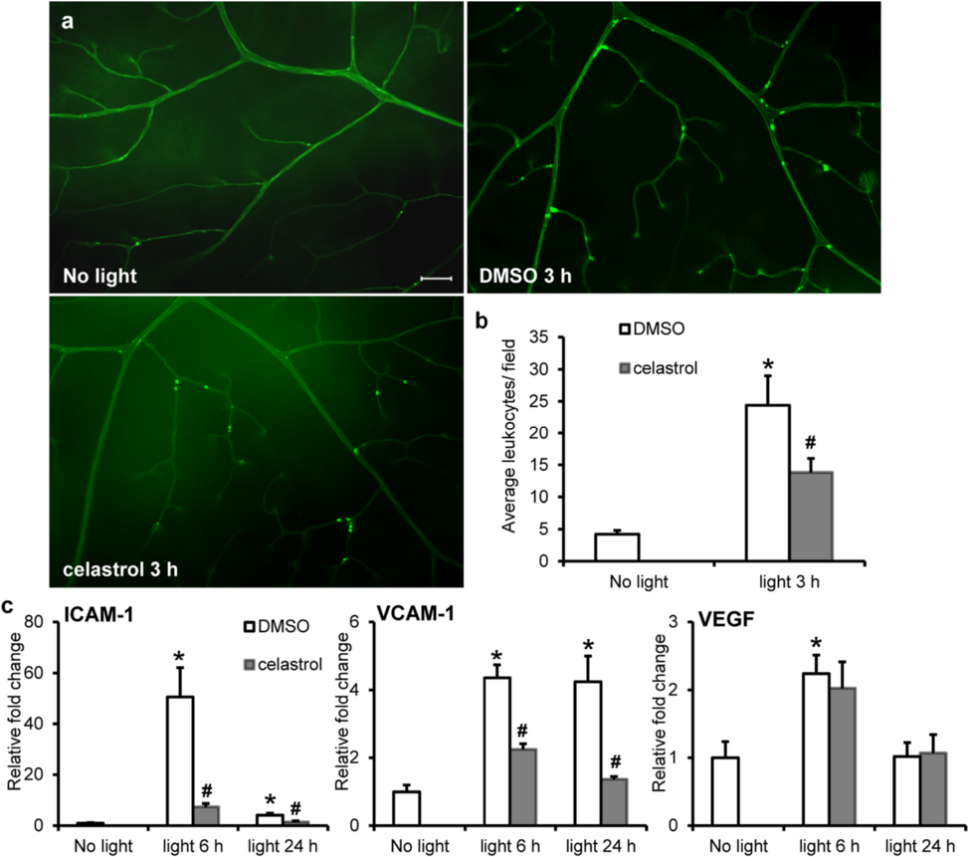
Celastrol suppressed retinal leukostasis and retinal expression of ICAM-1 and VCAM-1 in light-exposed mice. Dark-adapted BALB/c mice were exposed to bright light at 10,000 lx for 2 h after pretreatment with either DMSO or celastrol at 5 mg/kg bw. **a** Fluorescein-conjugated ConA labeling for adherent leukocytes in retinal vasculature was examined in mice without bright light exposure (*no light*) and 3 h after light exposure. Retinal flatmounts were made and observed under fluorescent microscope. **b** The number of loci with adherent leukocytes per field in each retina was counted (9–14 fields per retina, *n* = 4–6 retinas per group). *Scale bar*: 100 μm. **c** Retinas were collected 6 h and 1 day after light exposure and subjected to real-time PCR analyses for expression of ICAM-1, VCAM-1, and VEGF, respectively. Relative fold change against that from the mice without bright light exposure (*no light*) was presented. Data were expressed as mean ± S.E.M. (*n* = 4–6 per group). *Compared to that from no light, *p <* 0.05; ^#^ compared to that from DMSO, *p <* 0.05

Leukostasis is a pathological event closely associated with tissue inflammation [27]. Increased expression of proinflammatory genes in light-exposed retinas prompted us to further examine whether leukostasis is associated with light-induced retinal degeneration. Fluorescein-ConA labeling of adherent leukocytes in the retinal vasculature was examined after light exposure at 10,000 lx for 2 h. Adherent leukocytes were occasionally encountered in the retinal vasculatures from the mice unexposed to bright light; however, loci of adherent leukocytes were frequently observed in the retinal vasculature in light-exposed DMSO-treated mice 3 h after light exposure (Additional file 1: Figure S5). Much less adherent leukocytes were observed at 1 and 3 days after light exposure (Additional file 1: Figure S5). The effect of celastrol treatment on light-induced leukostasis was therefore assessed 3 h after light exposure. As shown in Fig. 6a, b, significantly decreased number of leukostasis loci in retinal vasculature was observed in light-exposed celastrol-treated mice compared to that from light-exposed DMSO-treated mice.

Leukostasis is mediated by intercellular adhesion molecule-1 (ICAM-1) [28], vascular adhesion molecule-1 (VCAM) [29], and vascular endothelial growth factor (VEGF) [30]. The expression of ICAM-1, vascular adhesion molecule-1 (VCAM-1), and VEGF was thus examined in the retinas. As shown in Fig. 6c, compared to that from the mice unexposed to bright light, transient and robust upregulation of retinal ICAM-1 expression was observed 6 h after light exposure in DMSO-treated mice. Retinal VCAM-1 expression exhibited increased expression at 6 h and 1 day after light exposure. Significant increase in retinal VEGF expression was observed at 6 h but not at 1 day after light exposure. In contrast, significantly decreased expression of ICAM-1 and VCAM-1 was observed in the retinas from light-exposed celastrol-treated mice at both 6 h and 1 day after light exposure. No significant difference in the retinal expression of VEGF was observed as a result of celastrol treatment in light-exposed mice. These results demonstrated that leukostasis was an early event associated with light-induced retinal degeneration, which could be significantly attenuated by celastrol treatment.

### Celastrol suppressed microglial activation and reactive gliosis in the retina in bright light-exposed mice

**Fig. 7:**
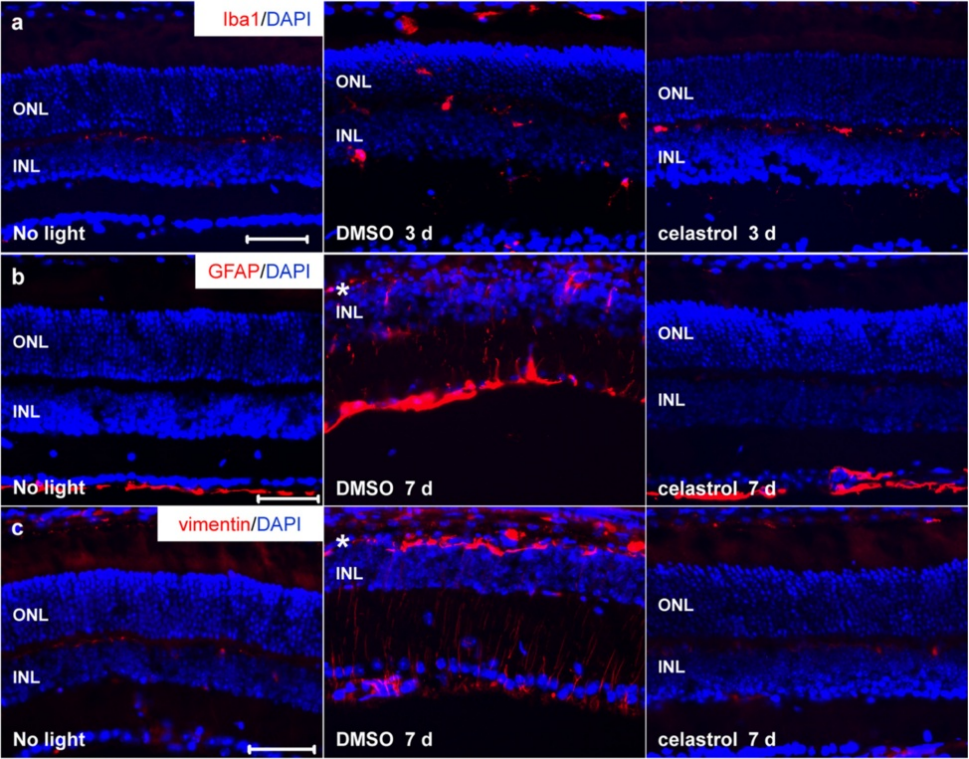
Celastrol suppressed microglial activation and gliosis in the retinas of bright light-exposed BALB/c mice. Dark-adapted BALB/c mice were exposed to bright light at 10,000 lx for 2 h after pretreatment with either DMSO or celastrol at 5 mg/kg bw. Cryosections made from eye cups collected 3 days after light exposure were subjected to IHC examination for the expression of Iba-1 (in *red*) (*n* = 3–5 per group) (**a**). Cryosections made from eye cups collected 7 days after light exposure were subjected to IHC examination of GFAP (in *red*) (*n* = 3–5 per group) (**b**) and vimentin (in *red*) (**c**) (*n* = 3–5 per group). DAPI counterstaining (in *blue*) was performed to visualize retinal gross morphology. *ONL* outer nuclear layer, *INL* inner nuclear layer. *Scale bar*: 50 μm

Microglial activation indicates sensing of retinal insults by microglia and contributes to tissue inflammation given the immunogenic nature of resident microglia, which could be identified by ectopic expression of microglial marker Iba-1. Moreover, gliosis reflects nonspecific changes in Müller glial cells in response to even subtle tissue damage, which is characterized by upregulated expression of intermediate filament protein glial fibrillary acid protein (GFAP) and vimentin. Thus, the effect of celastrol on expression of Iba-1, GFAP, and vimentin was further examined. As shown in Fig. 7a, compared to ramified expression restricted to the OPL of retinas in the mice without bright light exposure, enlarged amoeboid-shaped Iba-1-positive microglia were readily detected in INL, ONL, and subretinal spaces of retinas 3 days after bright light exposure in DMSO-treated mice. At 7 days after bright exposure, ONL was nearly diminished and ectopic expression of Iba-1 was not readily detected in light-exposed DMSO-treated mice (Additional file 1: Figure S6). In contrast, Iba-1 immunopositivity was evidently attenuated in the ONL and INL of celastrol-treated mice 3 days after bright exposure (Fig. 7a) and was not detected in the ONL of celastrol-treated mice 7 days after bright light exposure (Additional file 1: Figure S6). Moreover, as shown in Fig. 7b, the immunoreactivity of GFAP was prominently detected in the nerve fiber layer (NFL) in the retina of the mice unexposed to bright light. However, immunoreactivity of GFAP was readily observed in IPL, INL, and remaining ONL in the retina from DMSO-treated mice 7 days after light exposure at 10,000 lx for 2 h. Similar expression pattern was observed for the expression of vimentin, which became prominent throughout the damaged retina. In contrast, ectopic expression of GFAP and vimentin was not observed in the retina from light-exposed celastrol-treated mice (Fig. 7c). These results indicated celastrol treatment results in suppressed retinal microglial activation and gliosis in bright light-exposed BALB/c mice.

### Celastrol attenuated oxidative stress and suppressed the expression of proinflammatory genes in vitro

**Fig. 8:**
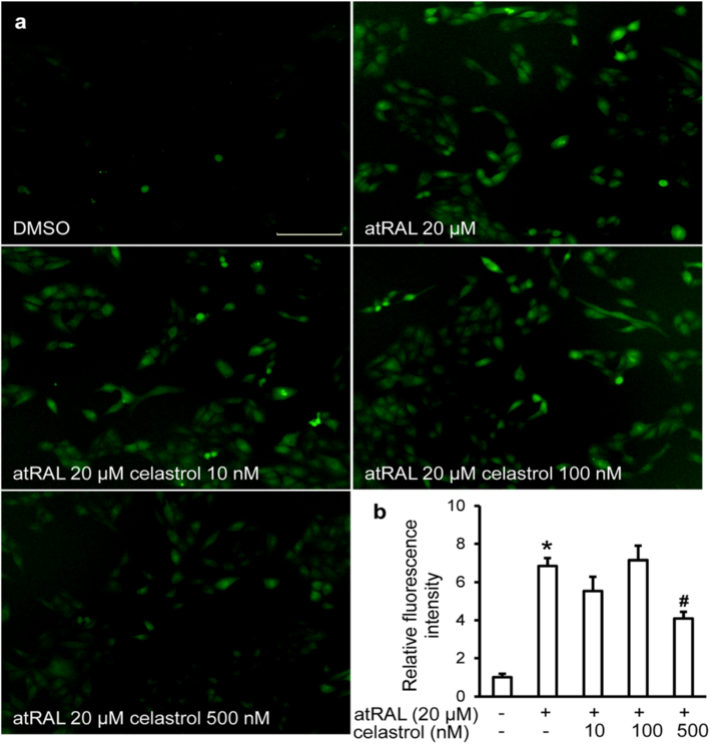
Celastrol reduced atRAL-induced oxidative stress in APRE19 cells. ARPE19 cells were pretreated with celastrol at indicated concentrations for 30 min prior to incubation of atRAL at 20 μM for 6 h. DCF-DA was then added to cells at 400 nM and incubated at 37 °C for 10 min. Cells was imaged for fluorescent signal indicative of ROS production under the same exposure setting using IncuCyte ZOOM (**a**). Fluorescence quantification was performed by IncuCyte ZOOM software under the setting of green fluorescence integrated intensity. Relative fluorescence intensities were calculated for statistical analyses (**b**). Data were expressed as mean ± S.E.M. (*n* = 4 per group). *Scale bar*: 150 μm. *Compared to that from vehicle-treated cells without atRAL and celastrol incubation, *p* < 0.05; ^#^compared to that from atRAL-stimulated vehicle-treated cells, *p* < 0.05

**Fig. 9:**
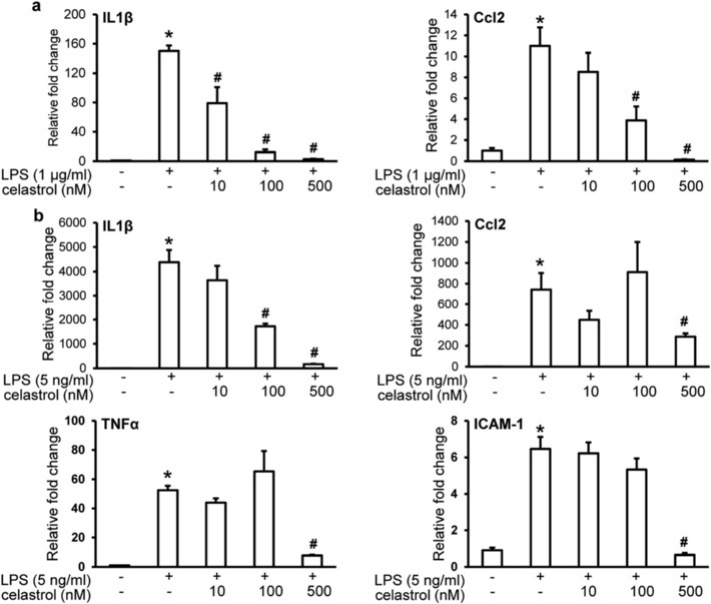
Celastrol suppressed the expression of proinflammatory genes in LPS-stimulated ARPE19 and RAW264.7 cells. ARPE19 (**a**) or RAW264.7 cells (**b**) were pretreated with celastrol at indicated concentrations for 30 min, which was followed by LPS incubation at concentrations of 1 μg/ml and 5 ng/ml, respectively. Cells were harvested 6 h later for RNA extraction. Real-time PCR analyses were subsequently performed to examine the expression of IL1β, Ccl2, TNFα, and ICAM-1. Data were expressed as mean ± S.E.M. (*n* = 4 per group). *Compared to that from vehicle-treated cells without LPS and celastrol incubation, *p* < 0.05; ^#^compared to that from the vehicle-treated cells stimulated with LPS, *p* < 0.05

To better characterize the pharmacological activities of celastrol, two cellular models were adopted. Firstly, ARPE19 cells, immortalized human RPE cells, were subjected to atRAL incubation to mimic light-induced retinal damage and oxidative stress in vivo [31]. ROS production in ARPE19 cells was then examined in the absence or presence of celastrol treatment. As shown in Fig. 8a, b, compared to that from vehicle controls, atRAL induced significant increase in ROS production, whereas significantly reduced ROS production was observed by celastrol treatment. Secondly, LPS-induced expression of proinflammatory genes was assessed in ARPE19 cells and mouse macrophage RAW264.7 cells in the presence of vehicle or celastrol treatment. Celastrol treatment resulted in significant suppression of LPS-induced expression of IL1β and Ccl2 in both APRE19 cells (Fig. 9a) and RAW264.7 cells (Fig. 9b). Additionally, although no endogenous expression and overt LPS-stimulated induction of TNFα and ICAM-1 was observed in ARPE19 cells, celastrol treatment significantly suppressed LPS-induced expression of TNFα and ICAM-1 in RAW264.7 cells (Fig. 9b). These results together not only confirmed the anti-inflammatory effect of celastrol in immune cells [18, 32] but also implied direct antioxidant and anti-inflammatory effects of celastrol in RPE cells.

## Discussion

The current study revealed that celastrol protected the retinas from morphological and functional impairment in bright light-exposed BALB/c mice. The retinal protection of celastrol was accompanied by remarkable suppression of light-induced photoreceptor apoptosis, ROS overproduction in RPE and photoreceptor cells, retinal expression of proinflammatory factors, leukostasis, retinal microglial activation, and gliosis.

Light-induced oxidative stress in the retina is causally associated with the pathogenesis of retinal degenerative disorders in patients and animal models [4, 33, 34]. Accumulated evidence supports an essential role for oxidative stress in RPE dysfunction that is primarily implicated in the pathogenesis of AMD. Oxidative damage to RPE is an early event in the development of AMD, and RPE dysfunction contributes to the loss of photoreceptors during the progression of AMD [35, 36]. The current study revealed rapidly occurring oxidative stress in RPE in bright light-exposed BALB/c mice. It is also noteworthy that oxidative stress in RPE was found to be more prominent and persistent than that detected in photoreceptor. The oxidative stress in RPE was readily detected 3 h after bright light exposure when only a few apoptotic photoreceptor cells were observed (Additional file 1: Figure S4 and Fig. 2). Prominent RPE oxidative stress was concomitant with increased photoreceptor cell death seen at 1 day after bright light exposure but was prior to massive photoreceptor cell death detected at 3 days after bright exposure (Additional file 1: Figure S4 and Fig. 2), providing in vivo evidence supporting that RPE oxidative stress is likely implicated in photoreceptor cell death. Most importantly, a remarkable effect of celastrol on suppressing light-induced oxidative stress in RPE was demonstrated (Fig. 3). Moreover, reduced level of ROS was observed in atRAL-stimulated ARPE 19 cells as a result of celastrol treatment, suggesting a direct effect of celastrol on attenuating oxidative stress in RPE cells (Fig. 8). These results partially explained the protective effects of celastrol against photoreceptor degeneration, and more importantly, they provided a potential pharmacological solution to alleviating oxidative stress in RPE.

Oxidative stress is noted as a principal mechanism of tissue stress, triggering inflammatory response in the retina [37–39]. The crosstalk between oxidative stress and inflammation is witnessed at molecular level as well. For instance, during retinal inflammation, oxidative stress is required for CCL2 production in response to inflammatory stimuli, which plays critical roles in promoting inflammation response by recruiting and activating various immune cells including monocytes, macrophages, and lymphocytes [40]. Celastrol treatment not only resulted in strong inhibition of light-induced oxidative stress in RPE but also significantly suppressed retinal expression of proinflammatory genes including Ccl2 in vivo. Moreover, activation of resident microglia plays important role in modulating inflammatory responses during the course of neurodegeneration. Activated microglia promotes neurodegeneration by secreting proinflammatory factors such as IL1β and TNFα [41, 42]. In human, retinal microglial activation is noted to be an event associated with photoreceptor death in several forms of retinal degenerative disorders [43]. Activated microglia has been increasingly recognized as hallmark pathology in degenerative retinas and contributes significantly to photoreceptor loss in mouse models manifesting light-induced retinal degeneration [11, 44, 45]. Moreover, therapies with inhibitory effects on microglial activation have been demonstrated to be neuroprotective in light-challenged retinas, emerging as new beneficial concepts for tackling related retinal degenerative disorders. For instance, it has been revealed that minocycline suppresses microglial activity in cultured microglial cells, attenuates microglial activation in the retinas, and protects against bright light-induced retinal degeneration in vivo [44]. Translocator protein (18 kDa) is highly expressed in reactive retinal microglia [46, 47], and a recent study has shown that it can be successfully targeted to counteract microglial activation and bright light-induced mouse retinal degeneration [45]. Inhibition of microglial activation is also protective against light-induced retinal damage in rats [48]. Of interest, it has been demonstrated that celastrol is equipped with direct suppressive effect on microglia activity in vitro. For example, it has been shown that in cultured mouse microglial BV-2 cells, celastrol inhibits LPS-induced upregulation of proinflammatory factors such as IL1β and TNFα [49]. Celastrol also suppresses double-strand RNA-stimulated microglial activation in mouse microglial MG-6 cells [50]. Here, we further showed that celastrol treatment suppressed bright light-induced retinal microglial activation in vivo (Fig. 7a). Given the neurotoxic nature of activated microglia, we reason that a direct suppressive activity of celastrol on microglial activation could contribute significantly to its protective effects against bright light-induced retinal degeneration.

Additionally, LPS-stimulated proinflammatory gene expression was significantly suppressed by celastrol treatment in ARPE19 and RAW264.7 cells (Fig. 9). However, LPS-induced TNFα expression was not observed in ARPE19 cells as that in RAW264.7 cells (Fig. 9b). These findings suggest that although both RPE and immune cells are likely the cellular targets of the anti-inflammatory actions of celastrol, they may play differential part in promoting inflammatory response under stress conditions.

Leucocytes play a crucial role in inflammation by interacting with endothelial cells, migrating to the sites of inflammation and releasing inflammatory cytokines. Under inflammatory conditions, endothelial cells are activated and express adhesion molecules including VEGF, ICAM-1, and VCAM-1 that cause leukocyte-endothelial cell interactions [51]. ICAM-1-mediated leukostasis has been identified as an early pathological event in the mouse model of diabetic retinopathy [52, 53]. Significantly enhanced expression of ICAM-1, VCAM-1, and VEGF in light-exposed retinas together with leukostasis in the retinal vasculature (Fig. 6 and Additional file 1: Figure S5) were observed prior to massive photoreceptor death (Fig. 2b), providing additional evidence supporting the notion that inflammation is associated with photoreceptor degeneration. However, future studies are required to delineate the link between leukostasis and photoreceptor death. Nonetheless, celastrol was shown here to significantly suppress the expression of ICAM-1 and VCAM-1 and decrease leukostasis lesions in retinal vasculature in bright light-exposed mice. These results may reinforce the concept of anti-inflammation in developing photoreceptor protective therapies. Moreover, LPS-induced ICAM-1 expression was not observed in APRE19 cells as that seen in RAW264.7 cells (Fig. 9b), and celastrol significantly suppressed LPS-induced ICAM-1 expression in RAW264.7 cells, further suggesting differential contributions of RPE and immune cells in inflammatory responses and the anti-inflammatory activity of celastrol.

Lastly, it is also noted celastrol does not have as significant an impact on steady-state retinal gene expression in the mice without bright light exposure (Additional file 1: Figure S7) as that in the mice exposed to bright light (Figs. 3b, 4 and 6c). Among eight genes analyzed, a marginal decrease of less than 30 % in the expression of VCAM-1 was observed 6 h but not 24 h after celastrol administration. A similar marginal decrease in VEGF expression was observed 24 h after celastrol administration. An about 1.9-fold increase in Ccl2 was observed in the retinas 6 h after celastrol administration. The rest of genes exhibited no significant changes.

## Conclusions

In summary, we have identified for the first time a potent effect of celastrol in protecting against light-induced massive loss of photoreceptors. The current findings also provide additional evidence supporting that oxidative stress in RPE and early activation of inflammatory response are implicated in the pathogenesis of light-induced photoreceptor degeneration, which can be significantly attenuated by celastrol administration. These findings thus support further evaluation of celastrol as a pharmacological candidate treating related retinal degenerative disorders.

### Availability of data and materials

The datasets supporting the conclusions of this article are included within the article.

## Additional file

Additional file 1:
**Figure S1**. Celastrol protected retinas from light-induced degeneration in BALB/c mice. **Figure S2**. Celastrol preserved retinal morphology in light-exposed BALB/c mice. **Figure S3**. Photoreceptor morphology in peripheral retina. **Figure S4**. Light stimulated ROS production in RPE. **Figure S5**. Light induced transient leukostasis in retinas. **Figure S6**. Retinal Iba-1 expression 7 days after bright light exposure in BALB/c mice. **Figure S7**. Expression of genes in celastrol-treated BALB/c mice without bright light exposure. (DOCX 7665 kb)

## Abbreviations

AMD: age-related macular degeneration
atRAL: all-*trans*-retinal
Ccl2: chemokine (C-C motif) ligand 2
ConA: concanavalin A
COX2: cyclooxygenase-2
DAPI: 4-6-diamidino-2-phenylindole
DCF-DA: 2′,7′-dichlorofluorescein diacetate
DHE: dihydroethidium
ERG: electroretinogram
GC: ganglion cells
GFAP: glial fibrillary acid protein
H&E: hematoxylin and eosin
HO-1: heme oxygenase 1
ICAM-1: intercellular adhesion molecule-1
IHC: immunohistochemistry
IL1β: interleukin 1β
INL: inner nuclear layer
IPL: inner plexiform layer
IS: inner segments
LPS: lipopolysaccharides
NFL: nerve fiber layer
OCT: optical coherence tomography
ONH: optic nerve head
ONL: outer nuclear layer
opsin M: mid-wavelength sensitive cone opsin
OS: outer segment
Rho: rhodopsin
ROS: reactive oxygen species
RP: retinitis pigmentosa
RPE: retinal pigment epithelium
TUNEL: TdT-mediated dUTP nick-end labeling
VCAM-1: vascular adhesion molecule-1
VEGF: vascular endothelial growth factor
