# Supplementary material for: Celastrol protects mouse retinas from bright light-induced degeneration through inhibition of oxidative stress and inflammation
Source: J Neuroinflammation. 2016 Feb 27;13:50. doi: 10.1186/s12974-016-0516-8 (PMC4769581; doi:10.1186/s12974-016-0516-8)
Supplement: Additional file 1: — Figure S1. Celastrol protected retinas from light-induced degeneration in BALB/c mice. Figure S2. Celastrol preserved retinal morphology in light-exposed BALB/c mice. Figure S3. Photoreceptor morphology in peripheral retina. Figure S4. Light stimulated ROS production in RPE. Figure S5. Light induced transient leukostasis in retinas. Figure S6. Retinal Iba-1 expression 7 days after bright light exposure in BALB/c mice. Figure S7. Expression of genes in celastrol-treated BALB/c mice without bright light exposure. (DOCX 7665 kb) [file 12974_2016_516_MOESM1_ESM.docx]

**Figure S1. Celastrol protected retinas from light-induced degeneration in BALB/c mice.** Dark-adapted BALB/c mice were exposed to white light at the intensity of 5,000 lux for 2 h (n=6-8 per group) (**a**), 10,000 lux for 30 min (n=4 per group) (**b**) or 10,000 lux for 2 h (n=6-8 per group) (**c**) after pretreatment with either vehicle control (DMSO) or celastrol at indicated doses. OCT imaging was carried out to record the retinal morphology 7 d after light exposure. Asterisk indicated diminished ONL. ONL, outer nuclear layer; INL, inner nuclear layer.

**Figure S2. Celastrol preserved retinal morphology** **in light-exposed BALB/c mice.** Dark-adapted BALB/c mice were exposed to light at the intensity of 5,000 lux for 2 h (**a**) and 10,000 lux for 30 min (**b**) after pretreatment with either vehicle control (DMSO) or celastrol at indicated doses. Eyes were enucleated 7 d after light exposure and paraffin sections were made and stained with H&E for retinal histological examination. Asterisk indicated disorganized and reduced length of outer/inner segments. ONL thickness was then quantified at 500 μm off optical nerve head in both superior and inferior retina for the mice without bright light exposure (No light) and those that had undergone light exposure at 5,000 lux for 2 h (n=3-4 per group) (**c**), 10,000 lux for 30 min (n=3-4 per group) (**d**). ONL, outer nuclear layer; INL, inner nuclear layer. Scale bar: 50 μm. * Compared to that from No light, *p*<0.05; ^#^ compared to that from DMSO, *p*<0.05.

**Figure S3**. Photoreceptor morphology in peripheral retina. Dark-adapted BALB/c mice were exposed to light at the intensity of 10,000 lux for 2 h after pretreatment with either DMSO or celastrol at 5 mg/kg bw. IHC examination of rhodopsin (Rho) and opsin M (in red) expression in the retinas were performed 7 d after light exposure along with DAPI counterstaining (in blue) in the cryosections. Peripheral retinal morphology was observed at 2500 μm of ONH (n=4 per group). ONL, outer nuclear layer; INL, inner nuclear layer. Scale bar: 50 μm.

**Figure S4.** **Light stimulated ROS production in RPE.** Dark-adapted BALB/c mice were exposed to light at 10,000 lux for 2 h and the retinal production of ROS was examined by DHE probe 3 h, 6 h, 1 d, 3 d and 7 d after light exposure along with that from the mice without light exposure (No light). DAPI staining of nuclei was performed to help visualize the retinal gross morphology. DAPI staining (in blue) and ROS signals (in red) were observed and recorded using fluorescence microscope (n=4 per group). RPE, retinal pigment epithelium; ONL, outer nuclear layer; INL, inner nuclear layer. Scale bar indicates 50 μm.

**Figure S5. Light induced transient leukostasis in retinas.** Dark-adapted BALB/c mice were exposed to light at 10000 lux for 2 h. Fluorescein-conjugated ConA labeling for adherent leukocytes in retinal vasculature was examined in mice without light exposure (No light), 3 h, 1 d and 3 d after light exposure. Retinal flatmounts were made and observed by fluorescent microscope (n=4-6 per group). Scale bar：100 μm.

**Figure S6. Retinal Iba-1 expression 7 d after bright light exposure in BALB/c mice.** Dark-adapted BALB/c mice were exposed to bright light at 10,000 lux for 2 h after pretreatment with either DMSO or celastrol at 5 mg/kg bw. Cryosections made from eye cups collected 7 d after light exposure were subjected to IHC examination for the expression of Iba-1 (in red) along with DAPI counterstaining (in blue) was performed (n=3-4 per group). Asterisk indicated diminished ONL. ONL, outer nuclear layer; INL, inner nuclear layer. Scale bar: 50 μm.

**Figure S7. Expression of genes in celastrol-treated BALB/c mice without bright light exposure.** BALB/c mice under normal laboratory light settings were subjected to treatment with either DMSO or celastrol at 5 mg/kg bw. Retinas were collected 6 h and 1 d after celastrol treatment and further processed for total RNA isolation, reverse transcription and real-time PCR analyses for expression of HO-1, IL1β, Ccl2, COX2, TNFα, ICAM-1, VCAM-1 and VEGF, respectively. After normalizing the expression to GAPDH, relative fold change of expression was calculated against that from the retinas collected from DMSO-treated mice. Data were expressed as mean±S.E.M (n=4-6 per group). * Compared to that from DMSO-treated mice, *p<*0.05.

**Figure S1**


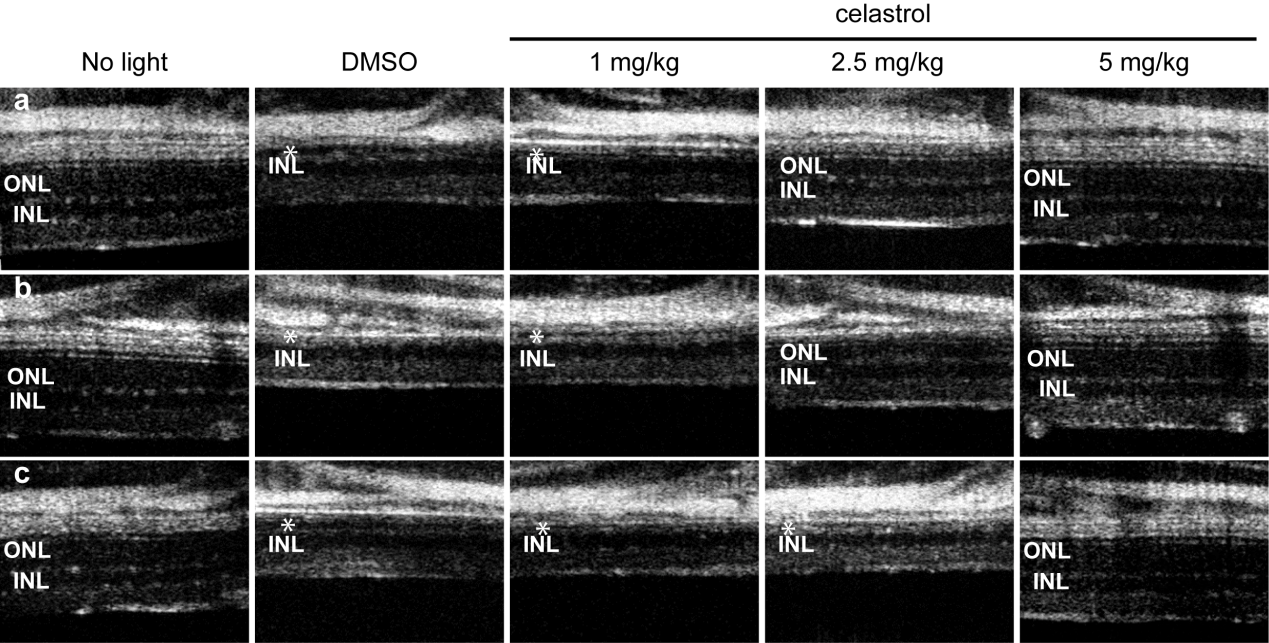


**Figure S2**

**
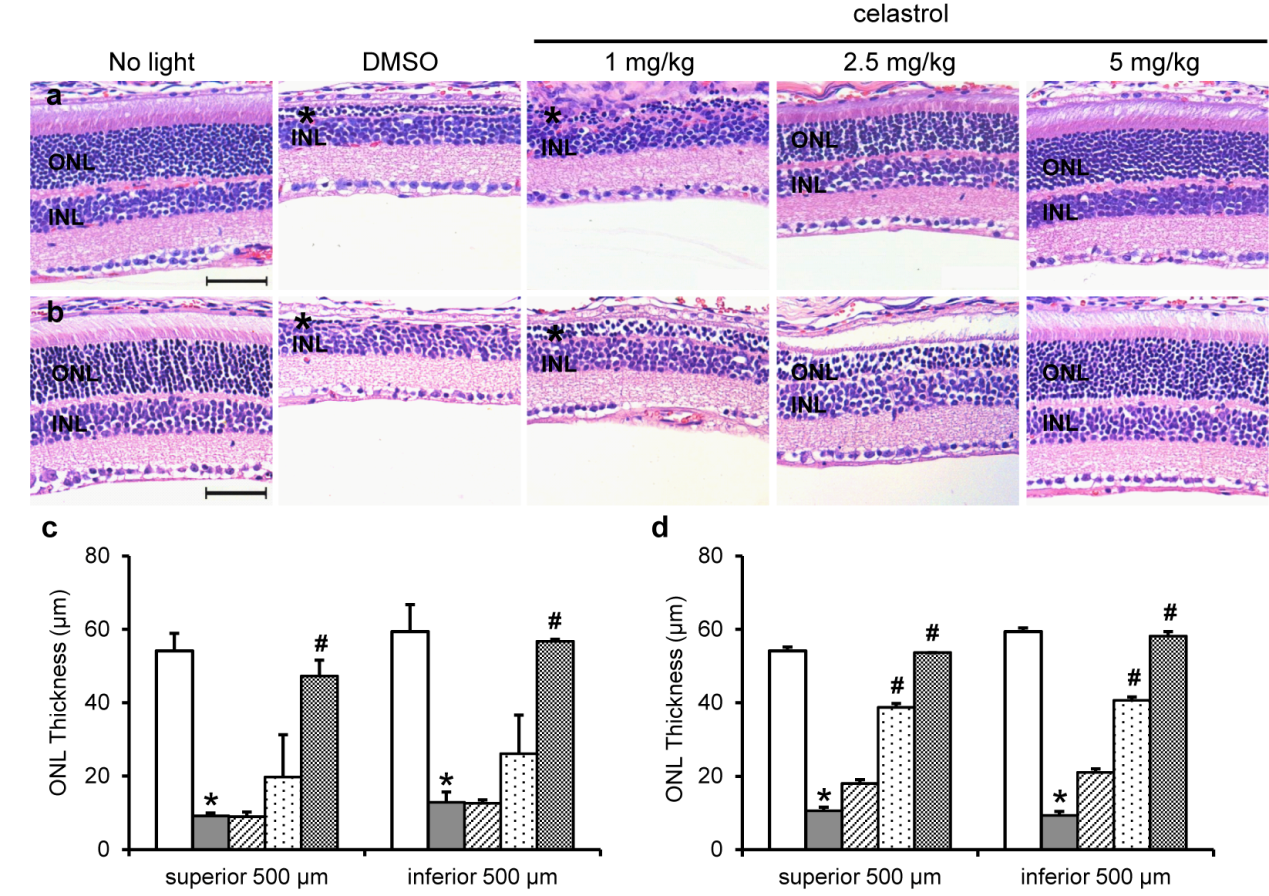
**

**Figure S3**

**
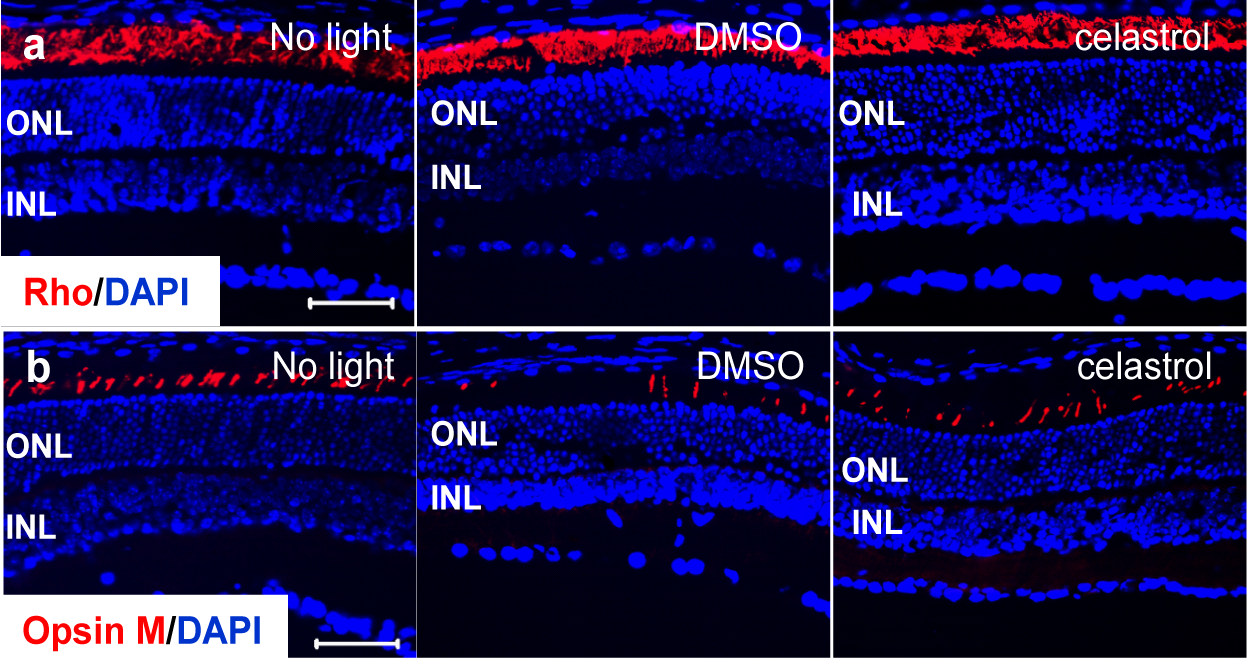
**

**Figure S4**

**
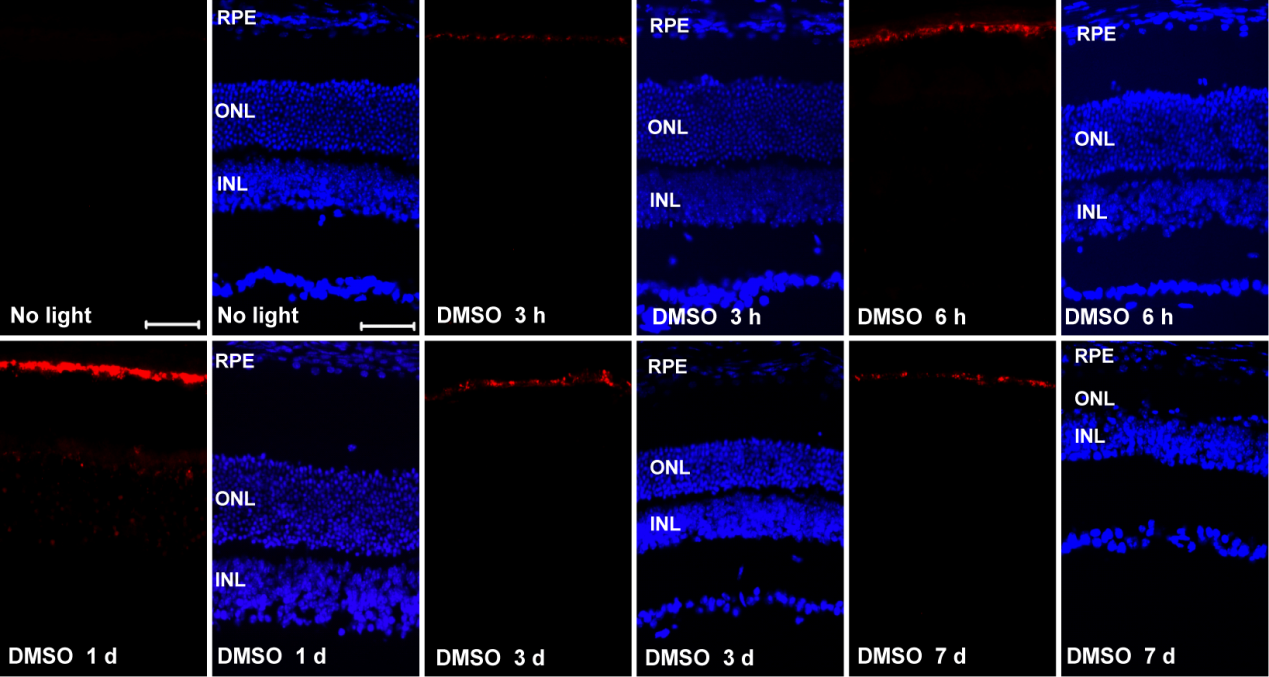
**

**Figure S5**

**
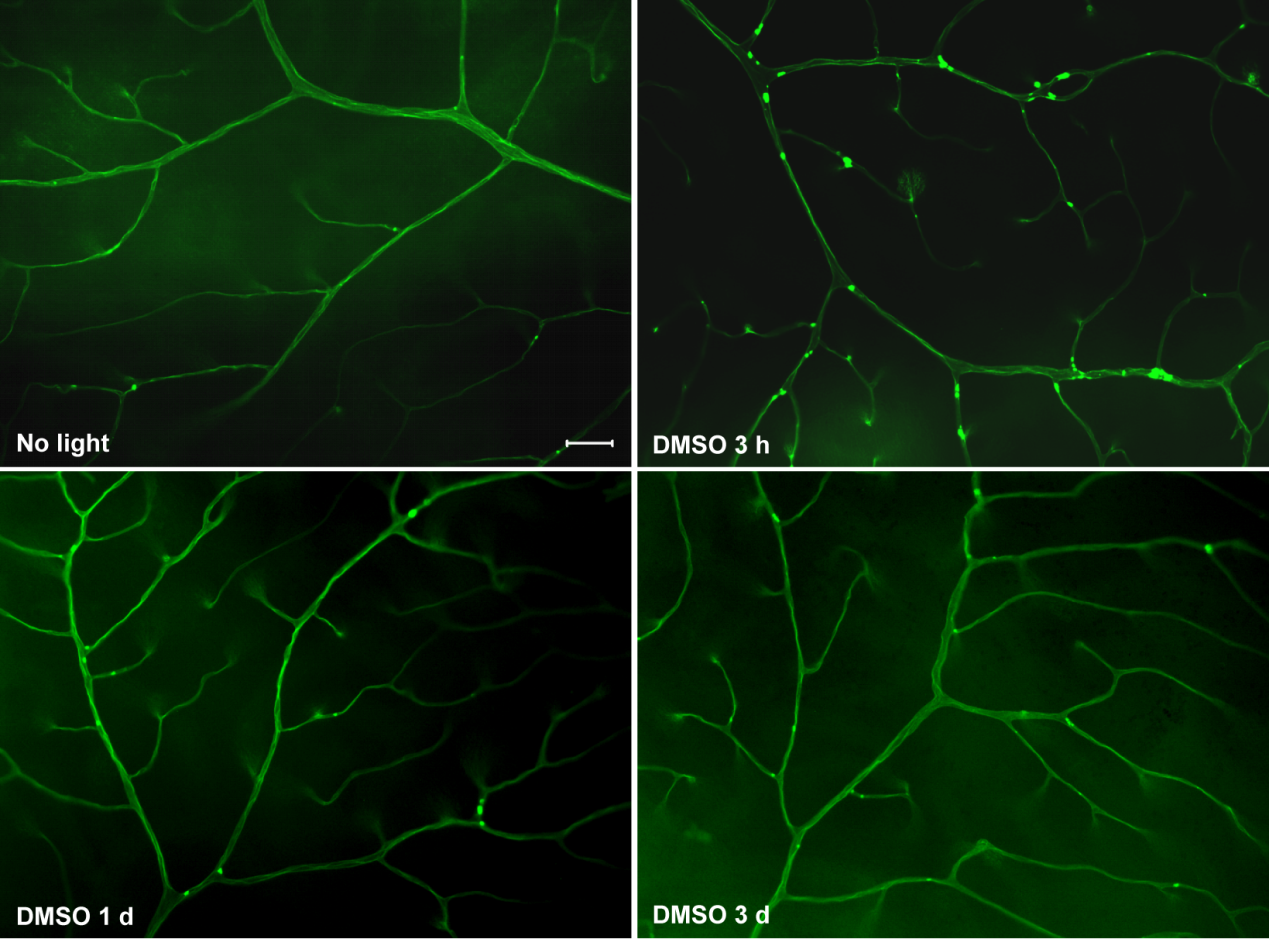
**

**Figure S6**

**
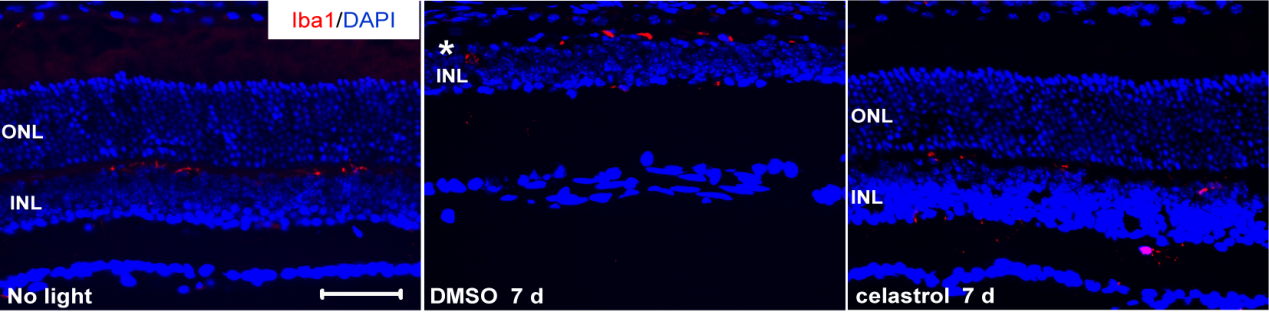
**

**Figure S7**

**
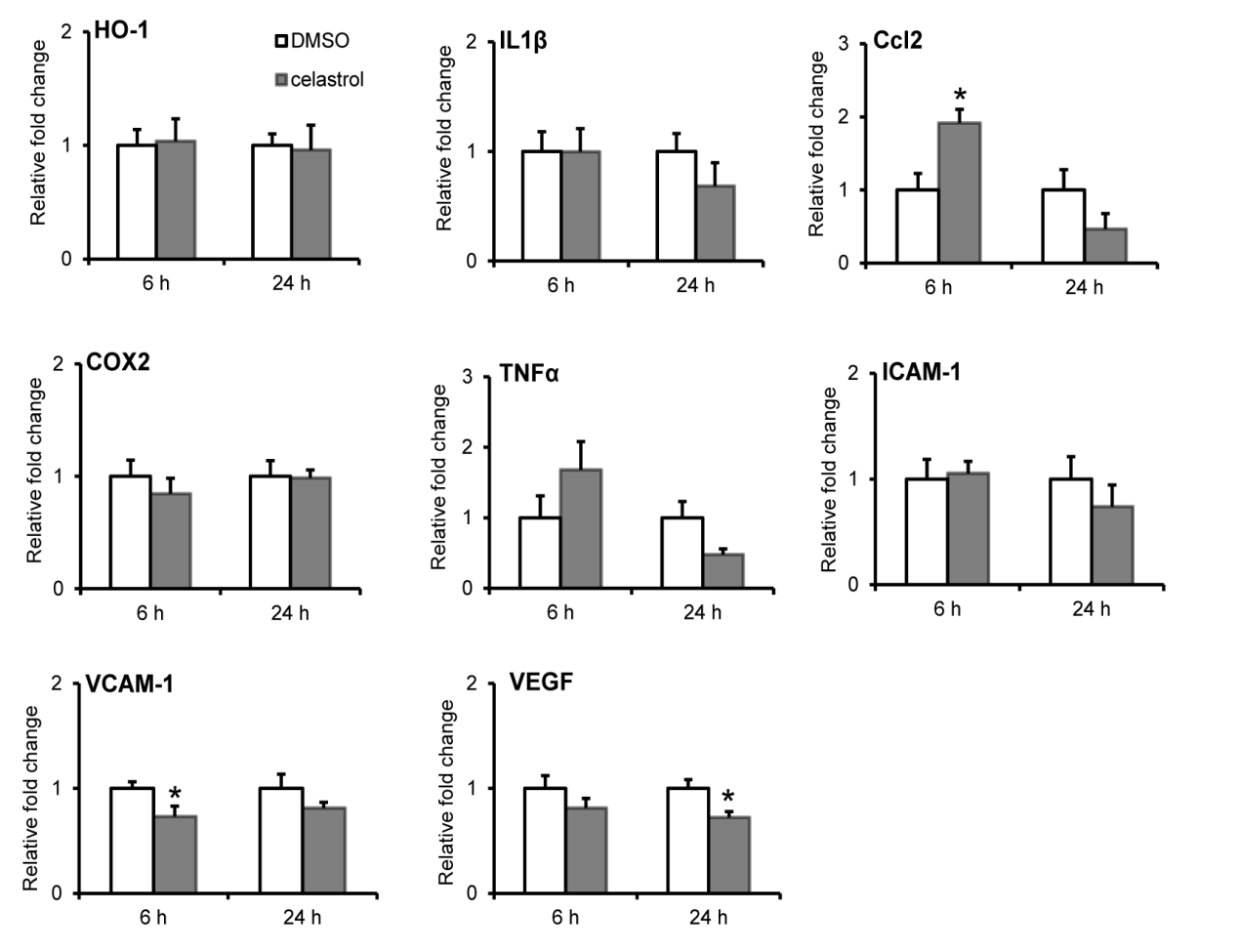
**
